# Voriconazole Activity Against *Pichia kudriavzevii*: Influence of Glucose Availability and Culture Medium on Growth, Biofilm Formation, and Antifungal Susceptibility

**DOI:** 10.3390/molecules31122161

**Published:** 2026-06-19

**Authors:** Marília Toledo Braga, Giulia Nicolle Jácome Cartaxo, Juliene Cristina da Silva Passos, Denilson Nogueira de Moraes, Carlos Alberto-Silva, Maricilia Silva Costa

**Affiliations:** 1 Instituto de Pesquisa & Desenvolvimento—IP&D, Universidade do Vale do Paraíba—UNIVAP, Av. Shishima Hifumi, 2911, São José dos Campos 12244-000, SP, Brazil; mariliatoledo.b@gmail.com (M.T.B.); giuliacartaxo@gmail.com (G.N.J.C.); passosjuliene@gmail.com (J.C.d.S.P.); dnmoraes@univap.br (D.N.d.M.); 2 Experimental Morphophysiology Laboratory, Natural and Humanities Science Center (CCNH), Federal University of ABC—UFABC, São Bernardo do Campo 09606-070, SP, Brazil; carlos.asilva@ufabc.edu.br

**Keywords:** *Pichia kudriavzevii* (*Candida krusei*), voriconazole (VRC), biofilm formation, RPMI-1640 medium, antifungal susceptibility testing, glucose metabolism

## Abstract

Invasive candidiasis remains a major cause of morbidity and mortality worldwide, with increasing relevance of non-*Candida albicans* species, particularly *Pichia kudriavzevii*, which is associated with high mortality and intrinsic resistance to fluconazole. This study evaluated the effect of voriconazole (VRC) on *P. kudriavzevii* growth, biofilm formation, and metabolic activity under different nutritional conditions. Planktonic growth and biofilm development were analyzed in Sabouraud dextrose broth (SDB), RPMI-1640, and RPMI-1640 supplemented with glucose (20 g·L^−1^). Antifungal activity was assessed by optical density (OD_570_) and XTT reduction assays, and biofilm morphology was examined by light microscopy. Glucose consumption was also determined during growth. VRC showed dose-dependent inhibition in SDB, reducing growth and biofilm metabolic activity by up to 94% and 98%, respectively. In contrast, in RPMI-1640, inhibition was significantly lower (≤27% growth and ≤77% biofilm reduction). Glucose supplementation partially restored antifungal susceptibility and increased biofilm metabolic activity. Growth kinetics confirmed VRC-induced delays in proliferation and impaired glucose utilization. These results demonstrate that VRC activity against *P. kudriavzevii* is strongly dependent on environmental nutrient availability, particularly glucose, which modulates fungal metabolism, biofilm development, and antifungal susceptibility, highlighting the importance of standardized antifungal susceptibility testing conditions and the role of metabolic state in azole efficacy.

## 1. Introduction

Invasive candidiasis remains a major cause of morbidity and mortality worldwide, particularly among hospitalized and immunocompromised patients, with candidemia associated with high mortality rates despite advances in antifungal therapy [1,2,3]. In recent years, epidemiological surveillance has shown a shift in species distribution, with a growing proportion of infections caused by non-albicans *Candida* species, including *Candida glabrata*, *Candida parapsilosis*, and *Pichia kudriavzevii* (formerly *Candida krusei*) [2,4,5,6]. Although *P. kudriavzevii* accounts for a relatively small percentage of bloodstream isolates (approximately 5%), its incidence has remained stable or increased in intensive care units, where it is frequently associated with critically ill patients and previous exposure to antifungal agents [7,8]. Importantly, infections produced by this species are associated with high mortality rates, ranging from 40% to 70% in severely ill or immunocompromised individuals, reflecting both patient severity and limited therapeutic options [3,4,9]. This epidemiological profile highlights the clinical relevance of *P. kudriavzevii* as an emerging opportunistic pathogen in healthcare settings, particularly in the context of invasive procedures, prolonged hospitalization and underlying comorbidities [5,10,11,12]. Notably, *P. kudriavzevii* exhibits intrinsic resistance to fluconazole, one of the most widely used azole antifungal agents for prophylaxis and treatment of candidiasis. It may also show variable susceptibility to other antifungal compounds within the azole class, reinforcing its clinical relevance in the context of antifungal-driven selection [7,13]. This resistance profile confers a selective advantage in healthcare environments with high antifungal exposure, thereby contributing to its persistence in clinical settings [3,4,14].

The mechanisms underlying antifungal resistance in *P. kudriavzevii* are considered multifactorial and may involve alterations in the ergosterol biosynthesis pathway, increased efflux pump activity and changes in antifungal target enzymes, mechanisms generally described in *Candida* species and suggested to contribute to reduced susceptibility in this species [9,15,16,17,18,19,20]. Despite intrinsic resistance to fluconazole, Voriconazole (VRC) has demonstrated in vitro activity against *P. kudriavzevii* and may represent a potential therapeutic choice in clinical scenarios, although its efficacy may be influenced by environmental and experimental conditions and should be interpreted alongside antifungal susceptibility testing and clinical context [3,4,14].

In addition to intrinsic resistance mechanisms, environmental and metabolic factors may influence antifungal susceptibility and biofilm-associated phenotypes in *P. kudriavzevii*. Among these, glucose availability has been reported to modulate biofilm development and fungal physiology in *Candida* species, potentially affecting susceptibility to antifungal agents, including azoles [12,13,14]. However, the impact of these conditions on VRC activity on *P. kudriavzevii* under different culture media remains insufficiently understood. Culture medium composition is a key determinant of antifungal susceptibility testing (AFST), as it directly affects fungal growth, metabolism, and antifungal activity, with consequent impact on Minimum Inhibitory Concentration (MIC) determination and reproducibility [21,22,23]. Variations in pH, glucose concentration and buffering capacity can significantly modify antifungal responses, reducing interlaboratory comparability and reinforcing the need for rigorous methodological standardization [9,24,25]. Therefore, CLSI and EUCAST recommend microdilution using RPMI-1640 medium as the reference method due to its chemically defined composition and superior reproducibility [26,27,28]. In contrast, Sabouraud Dextrose Broth (SDB), although widely used for fungal isolation, is not appropriate for quantitative AFST because its acidic pH and high glucose content can alter fungal physiology and antifungal pharmacodynamics, leading to increased variability in MIC endpoints [24,29]. Surveillance studies, including SENTRY and ARTEMIS, based on RPMI-1640 methodologies, have reported consistent MIC values for *P. kudriavzevii*, with MIC50 values around 0.25–0.5 µg·mL^−1^ VRC and MIC_90_ values generally ranging from 1 to 2 µg·mL^−1^, supporting its in vitro activity despite intrinsic fluconazole resistance [30,31].

Recently, it has been observed that the composition of dual-species biofilms formed by *C. albicans* and *P. kudriavzevii* can be significantly influenced by the culture medium [32]. When RPMI-1640 is used, *C. albicans* predominates over *P. kudriavzevii*, whereas in the presence of SDB, a relative increase in *P. kudriavzevii* development was observed, indicating a medium-dependent shift in species distribution rather than an intrinsic species preference. In addition, environmental and nutritional conditions, including culture medium composition, can influence biofilm architecture, adhesion and metabolic activity in *Candida* species [33,34]. Thus, despite the recognized influence of environmental conditions on Candida physiology, the relationship between culture medium composition, glucose availability, and VRC susceptibility in *P. kudriavzevii* remains insufficiently understood. The objective of this study was to evaluate the inhibitory activity of VRC against *P. kudriavzevii* under distinct culture conditions, comparing RPMI-1640 and SDB, which differ in glucose content, pH and buffering capacity.

## 2. Materials and Methods

### 2.1. Yeast Strain and Growth Conditions

*P. kudriavzevii* (ATCC 6258) was plated on Sabouraud dextrose agar (Merck, Darmstadt, Hesse, Germany) and incubated in atmospheric air for 48 h, at 37 °C. After that, a sample of the colonies was removed and suspended in a saline sterilized solution (0.85% NaCl). Cell density was adjusted using a Neubauer chamber. Unless otherwise specified, all experiments were performed using eight independent biological replicates (*n* = 8), each analyzed in technical triplicate. The mean of technical replicates was used for statistical analysis. When different experimental designs were applied, the corresponding numbers of biological and technical replicates are indicated in the corresponding experimental section.

### 2.2. Biofilm Formation by P. kudriavzevii

Cell suspensions (25 µL) of *P. kudriavzevii* (10^7^ cells·mL^−1^) were added to a 96-well microtiter plate, containing Sabouraud-dextrose broth (SDB) (peptone 10 g·L^−1^, dextrose 40 g·L^−1^; pH 5.6 ± 0.2) (Merck, Darmstadt, Hesse, Germany), RPMI-1640 medium (buffered with 0.165 mol·L^−1^ MOPS, pH adjusted to 7.0) (Sigma, St. Louis, MO, USA) or RPMI-1640 supplemented with glucose (20 g·L^−1^) in the presence of VCR (ranging from 0.125 to 2 µg·mL^−1^), in a final volume of 200 µL. The plates were incubated for 24 h at 37 °C and after this period, the supernatant (containing cells in suspension) was carefully collected and reserved to determine cell growth; and the biofilms produced were washed once with PBS (200 µL) to remove non-adherent cells and traces of the medium. Following, metabolic activity of biofilms produced was determined by a metabolic assay based on the reduction of the sodium salt of XTT (2,3-bis(2-methoxy-4-nitro-5-sulfophenyl)-2H-tetrazolium-5-carboxanilide) (Molecular Probes, Eugene, OR, USA). Aliquots (100 µL) of a mixture of XTT and Menadione (0.4 mM) (Sigma, St. Louis, MO, USA) in a 3:1 ratio prepared in PBS was added to each well and incubated for 2 h at 37 °C. The reaction product was measured by absorbance at 490 nm (OD_490_) in a spectrophotometer Synergy HT Multi-Detection Microplate Reader (Bio-Tek, Winooski, VT, USA). The data obtained were expressed as percentage (%) of metabolic activity determined in relation to the control group (cells incubated in the absence of VCR).

### 2.3. Effect of VRC on 24 h Preformed Biofilms of P. kudriavzevii

Cell suspensions (40 µL) of *P. kudriavzevii* at cell density of 10^7^ cells·mL^−1^ were added to a 96-well microtiter plate containing SDB, RPMI-1640 or RPMI-1640 supplemented with glucose (20 g·L^−1^), in a final volume of 200 µL and incubated for 24 h (37 °C) to produce biofilm. Then, the content of wells was removed and the wells were washed once with PBS (100 µL) to remove non-adherent cells. Following, fresh SDB, RPMI-1640 or RPMI-1640 supplemented with glucose (20 g·L^−1^), containing different concentrations of VRC (0.125 to 2 µg·mL^−1^), was added to each well in a final volume of 200 µL and incubated for an additional 24 h at 37 °C. After this period, the supernatant (containing cells in suspension) was carefully collected and reserved to determine cell growth; and the wells were washed once with PBS (200 µL) in order to remove non-adherent cells and traces of medium, and metabolic activity of biofilms produced was determined using XTT metabolic assay, according to methodology described in Section 2.2. The data obtained were expressed as percentage (%) of metabolic activity determined in relation to the control group (no VCR).

### 2.4. P. kudriavzevii Growth

Supernatants from assays described in 2.2 and 2.3 sections were used to determine cell growth by recording the optical density at 570 nm (OD_570_) using the spectrophotometer SynergyHT Multi-Detection Microplate Reader (Bio-Tek, Winooski, VT, USA) and the data obtained were expressed as percentage (%) of growth in relation to the control group (cells incubated in the absence of VCR). For the purposes of this study, MIC50 was operationally defined as the lowest concentration of VRC resulting in a 50% reduction in growth or metabolic activity relative to the untreated control.

### 2.5. Morphological Analyses of Biofilms

The morphology of biofilms produced after different treatments was monitored by inverted light microscopy (Nikon Eclipse TS 100, Tokyo, Japan) and the images were captured with a Moticam 580 digital camera system (Motic, Xiamen, China) coupled to the photomicroscope and to a microcomputer using the software Motic Images Plus 2.0 (Motic, Xiamen, China).

### 2.6. P. kudriavzevii Viability Assay by Plating Supernatant

Aliquots of supernatant (25 µL) from assays described in Section 2.2 and Section 2.3, containing different concentrations of VRC (0.125 to 2 µg·mL^−1^), were plated on Sabouraud dextrose agar (Merck, Darmstadt, Hesse, Germany) and incubated in atmospheric air, at 37 °C for 24 h. Then, colony growth was observed and images were captured by a smartphone camera (Samsung Galaxy S24, Samsung Electronics, Gyeonggi, Republic of Korea).

### 2.7. Time-Course Growth Assay of P. kudriavzevii

Cell suspensions at 10^5^ cells·mL^−1^ of *P. kudriavzevii* were added to a 24-well microtiter plate (Corning Incorporated, Corning, NY, USA), containing SDB, in the presence of VCR ranging from 0.01 to 0.2 µg·mL^−1^, in a final volume of 1 mL and incubated aerobically at 37 °C for 48 h. Growth was monitored every 2 h by recording the optical density at 570 nm (OD_570_) using the spectrophotometer SynergyHT Multi-Detection Microplate Reader (Bio-Tek, Winooski, VT, USA). The experiments were performed in duplicate in four independent biological experiments and the mean of technical replicates was used for analysis.

### 2.8. Glucose Determination

Glucose concentration was determined by high-performance liquid chromatography (HPLC) using a Shimadzu Prominence-i LC-2030C system (Shimadzu, Tokyo, Japan) equipped with a diode array detector (DAD). Chromatographic separation was performed on a Shim-pack GIST NH_2_ column (4.6 × 250 mm, 5 μm particle size). The mobile phase consisted of acetonitrile and ultrapure water (Milli-Q grade) at an 85:15 (*v*/*v*) ratio. The mobile phase was prepared daily and filtered through a 0.22 μm membrane filter before use. The flow rate was maintained at 0.8 mL min^−1^, and the column temperature was set at 30 °C. Calibration curves for external quantification were prepared using an analytical D-glucose standard dissolved in ultrapure water, covering a linear range of 0.4–2.4% (*w*/*v*). Blank mobile-phase injections were routinely performed to verify the absence of interfering peaks at the glucose retention time. Chromatographic data acquisition, integration, and processing were performed using LabSolutions software (version 5.82; Shimadzu Corporation, Kyoto, Japan).

For glucose quantification, samples obtained from time-course growth assays conducted in the absence or presence of VRC (0.05 and 0.1 μg·mL^−1^) were collected after 12, 20, and 28 h of incubation. Samples were filtered through a 0.22 μm membrane filter prior to injection. The injection volume was 10 μL, and detection was performed at 190 nm. Experiments were carried out in duplicate technical replicates within three independent biological experiments. For statistical analysis, the mean value of the technical replicates was used. Samples were filtered through a 0.22 μm membrane filter prior to injection. The injection volume was 10 μL, and detection was carried out at 190 nm. The experiments were performed in duplicate in three independent biological experiments and the mean of technical replicates was used for analysis.

### 2.9. Statistical Analyses

Statistical differences were evaluated using one-way analysis of variance (ANOVA), followed by the Tukey–Kramer post hoc test. The numbers of biological and technical replicates used in each experiment are described in the corresponding sections. *p* values < 0.05 were considered statistically significant. Graphs were generated and statistical analyses were performed using OriginPro 8.5 (OriginLab Corporation, Northampton, MA, USA).

## 3. Results

### 3.1. Effect of VRC on Growth and Biofilm Formation Under Different Culture Media

**Figure 1:**
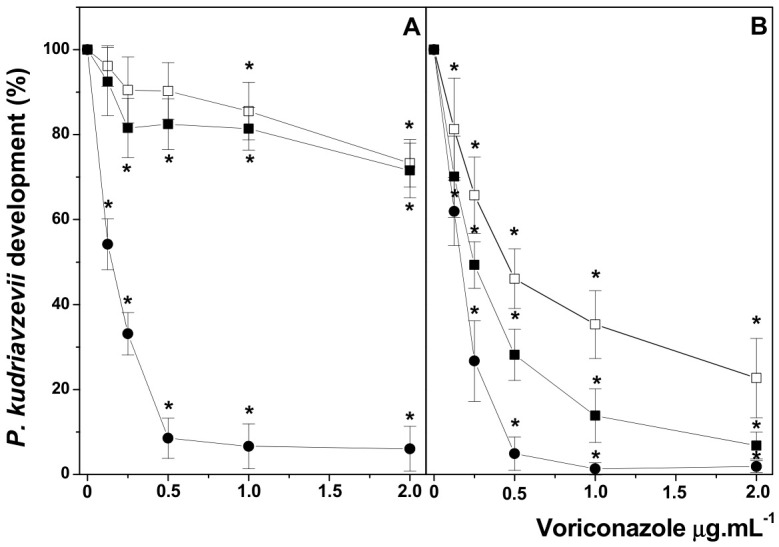
Effect of voriconazole (VRC) on the growth and biofilm formation by *P. kudriavzevii*. (**A**) Planktonic growth and (**B**) biofilm formation by *P. kudriavzevii* in the presence of increasing concentrations of VRC. Cell suspensions were cultured for 24 h in SDB (•), RPMI-1640 (□) or RPMI-1640 supplemented with glucose (■), as described in Section 2. *P. kudriavzevii* growth was quantified by measuring optical density at 570 nm (OD570), whereas biofilm metabolic activity was evaluated using the XTT reduction assay. Results are presented as mean ± standard deviation (SD) and expressed as percentage (%) relative to the untreated control group (100%). Statistical significance was determined compared with the respective control group (* *p* < 0.05).

Initially, the effect of VRC on *P. kudriavzevii* development was evaluated using either SDB or RPMI-1640 medium (Figure 1). A pronounced inhibition of *P. kudriavzevii* growth by VRC was observed in cells grown in the presence of SDB (Figure 1A). Growth inhibition of 46, 67, and 94% was observed with 0.125, 0.25, and 2 µg·mL^−1^ VRC, respectively. At the same time, biofilm formation was evaluated, and a similar inhibition profile was observed, with inhibitions of 38, 73, and 98% at 0.125, 0.25, and 2 µg·mL^−1^ VRC, respectively (Figure 1B). In addition, a positive correlation (r = 0.98) between growth and biofilm formation by *P. kudriavzevii* was observed with increasing VRC concentrations, and a MIC50 value of approximately 0.2 µg·mL^−1^ VRC was determined for both growth and biofilm formation.

However, when *P. kudriavzevii* development was evaluated using RPMI-1640 medium, the inhibition profile produced by VRC was markedly altered. Growth reductions of 15 and 27% were observed with 1 and 2 µg·mL^−1^ VRC, respectively (Figure 1A). However, when analyzing biofilm formation in RPMI-1640 medium, inhibitions of 34, 65, and 77% were observed with 0.25, 1, and 2 µg·mL^−1^ VRC, respectively (Figure 1B). These results indicate that biofilm formation by *P. kudriavzevii* in RPMI-1640 medium may be more susceptible to VRC treatment than planktonic cell growth; consequently, no correlation between growth and biofilm formation was observed. A MIC50 value of approximately 0.5 µg·mL^−1^ VRC was determined for biofilm formation by *P. kudriavzevii*.

Additionally, the inhibition profile produced by VRC was evaluated during *P. kudriavzevii* development in RPMI-1640 supplemented with glucose. RPMI-1640 supplemented with glucose (20 g·L^−1^) was used to more closely resemble the high-glucose conditions of SDB, permitting comparison of VRC activity under different carbon availability conditions. No significant change was observed in the inhibition of *P. kudriavzevii* growth by VRC. However, the addition of glucose (20 g·L^−1^) to RPMI-1640 increased the inhibitory effect of VRC on biofilm formation. Biofilm formation in RPMI-1640 supplemented with glucose showed inhibitions of 51, 86, and 93% with 0.25, 1, and 2 µg·mL^−1^ VRC, respectively (Figure 1B).

These results demonstrate that the inhibitory activity of VRC against *P. kudriavzevii* development can be substantially modified by the culture medium used and that glucose concentration is an important factor modulating the effect of VRC. A particularly important point to highlight is that, in all experiments performed, the development of *P. kudriavzevii* was, as expected, greater in SDB than in RPMI-1640 medium. In this work, yeast proliferation was measured based on absorbance of the growth medium, and the absorbance values determined after 18 h of growth in SDB, RPMI-1640, and RPMI-1640 supplemented with glucose were 1.1 ± 0.03, 0.29 ± 0.03, and 0.23 ± 0.06, respectively. In contrast, the metabolic activity values associated with biofilm formation were 0.38 ± 0.05, 0.22 ± 0.07, and 0.40 ± 0.11 in biofilms produced in SDB, RPMI-1640, and RPMI-1640 supplemented with glucose, respectively. These results demonstrate that glucose supplementation of RPMI-1640 increases biofilm metabolic activity to levels comparable to those observed in SDB.

### 3.2. Effect of VRC on Cell Viability

**Figure 2:**
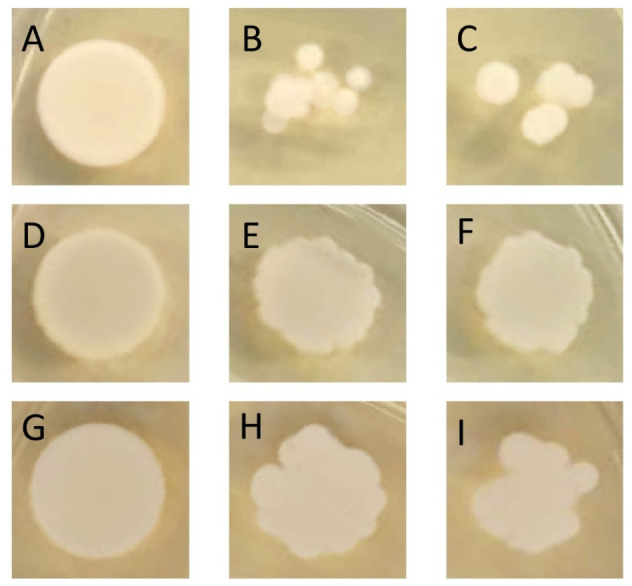
Effect of VRC on cell viability of *P. kudriavzevii* cells grown under different culture conditions. *P. kudriavzevii* cell suspensions were cultured for 24 h in SDB (**A**–**C**), RPMI-1640 (**D**–**F**) or RPMI-1640 supplemented with glucose (**G**–**I**), in the absence of VRC (**A**,**D**,**G**) or in the presence of 1 µg·mL^−1^ VRC (**B**,**E**,**H**) or 2 µg·mL^−1^ VRC (**C**,**F**,**I**). After incubation, aliquots of the culture supernatants were plated onto Sabouraud dextrose agar plates and incubated for an additional 24 h (37 °C) to evaluate cell viability based on colony growth. Representative images of colony formation are shown.

Cell viability after 18 h of exposure to 1 and 2 µg·mL^−1^ VRC was evaluated by colony formation on agar plates (Figure 2). Compared with the control group, VRC treatment markedly reduced the number of viable cells in cultures grown in SDB medium. VRC also decreased the viability of cells grown in RPMI-1640 and RPMI-1640 supplemented with glucose; however, this effect was substantially less pronounced than that observed in cells grown in SDB.

### 3.3. Morphological Characterization of Biofilm Formation and VRC-Induced Structural Changes Under Different Culture Conditions

**Figure 3:**
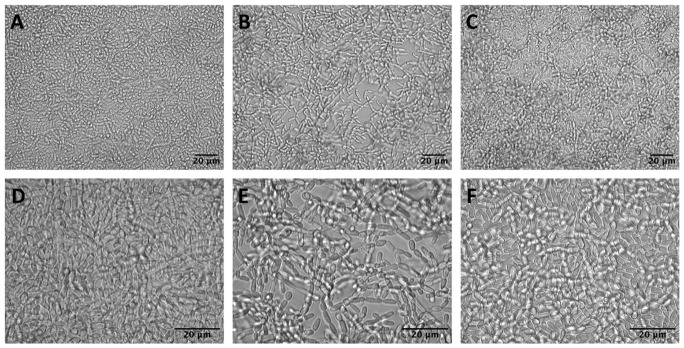
Morphological characterization of biofilm formation by *P. kudriavzevii* under different culture conditions. Biofilms were formed for 24 h in SDB (**A**,**D**), RPMI-1640 medium (**B**,**E**) or RPMI-1640 supplemented with glucose (**C**,**F**), as described in Section 2. Representative images were obtained by inverted light microscopy at magnifications of 20× (**A**–**C**) and 40× (**D**–**F**). Scale bar = 20 µm.

The structure of biofilms produced by *P. kudriavzevii* was also evaluated under different culture conditions (Figure 3). Biofilms formed in SDB appeared more robust, with a higher density of adhered cells (Figure 3A,D). In contrast, biofilms produced in RPMI-1640 exhibited lower cell density, although filamentous forms were observed (Figure 3B,E). Moreover, biofilms formed in RPMI-1640 frequently detached from the plate surface during the washing steps. Notably, biofilms formed in RPMI-1640 supplemented with glucose exhibited increased cell density, resembling those formed in SDB (Figure 3C,F), consistent with the enhanced metabolic activity observed in these biofilms (Figure 1). Taken together, these findings highlight the importance of glucose availability for both cell adhesion and the metabolic activity of cells within the biofilm structure.

**Figure 4:**
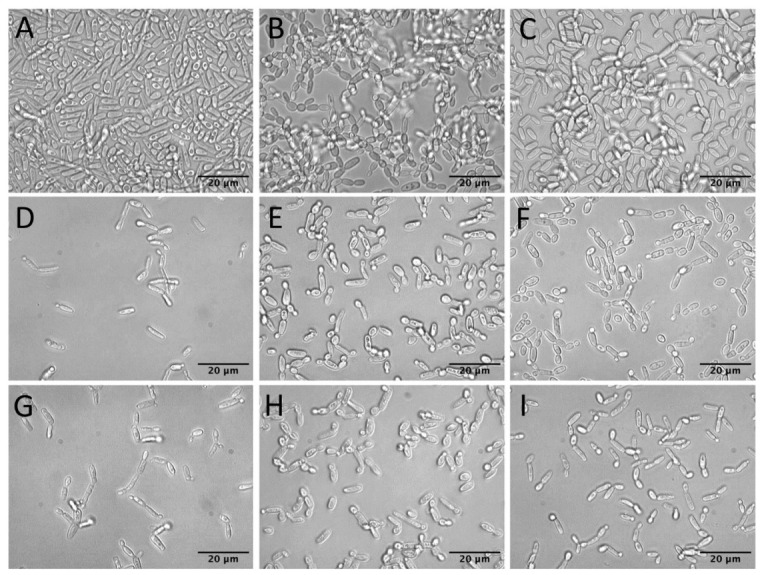
Effect of voriconazole (VRC) on the morphology of *P. kudriavzevii* biofilms under different culture conditions. Biofilms were formed for 24 h in SDB (**A**,**D**,**G**), RPMI-1640 medium (**B**,**E**,**H**) or RPMI-1640 supplemented with glucose (**C**,**F**,I), as described in Section 2. Biofilms were developed in the absence of VRC (**A**–**C**) or in the presence of 1.0 µg·mL^−1^ VRC (**D**–**F**) or 2.0 µg·mL^−1^ VRC (**G**–**I**). Representative images were obtained by inverted light microscopy at 40× magnification. Scale bar = 20 µm.

Figure 4 corroborates the findings presented in Figure 1, demonstrating that the inhibitory effect of VRC against *P. kudriavzevii* depends on the culture medium used for biofilm formation. A marked reduction in the number of cells within the biofilm structure was observed in biofilms formed in SDB in the presence of either 1 or 2 µg·mL^−1^ VRC (compare Figure 4A,D,G). Compared with the control group, biofilms formed in RPMI-1640 or RPMI-1640 supplemented with glucose were also affected by VRC treatment, although to a lesser extent (Figure 4B,C,E,F,H,I).

### 3.4. VRC Effects on Preformed 24 h Biofilms: Metabolic Activity, Growth and Structural Changes

**Figure 5:**
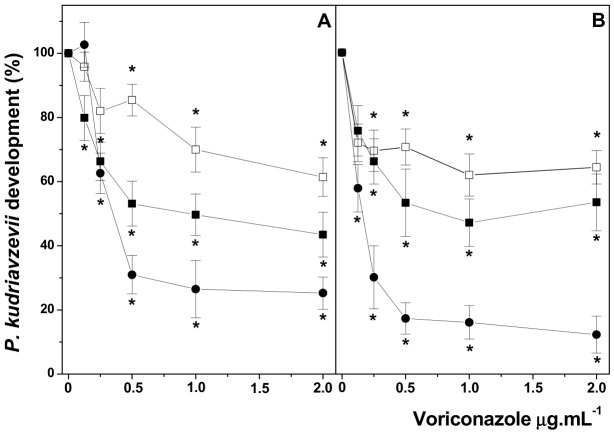
Effect of VRC on the planktonic growth and metabolic activity of 24 h *P. kudriavzevii* biofilms under different culture conditions. (**A**) Planktonic growth and (**B**) metabolic activity of preformed 24 h biofilms exposed to increasing concentrations of VRC. Biofilms were initially formed for 24 h in Sabouraud dextrose broth (SDB) (•), RPMI-1640 medium (□), or RPMI-1640 supplemented with glucose (■), followed by an additional 24 h incubation in the presence of VRC, as described in Section 2. Planktonic growth was quantified by measuring optical density at 570 nm (OD570), whereas biofilm metabolic activity was determined using the XTT reduction assay. Results are expressed as mean ± standard deviation (SD) and are presented as percentage (%) relative to the untreated control group (100%). * *p* < 0.05 compared with the respective control group.

The effect of VRC on 24 h preformed biofilms produced in SDB, RPMI-1640, and RPMI-1640 supplemented with glucose is shown in Figure 5. Biofilms formed for 24 h in SDB and subsequently exposed to VRC for an additional 24 h exhibited reductions of 71% and 90% in metabolic activity at VRC concentrations of 0.25 and 2 µg·mL^−1^, respectively (Figure 5B). During this additional incubation period, cells detach from the biofilm structure, disperse into the surrounding medium, continue to proliferate, and can be quantified by measuring the absorbance of the culture medium. Under these conditions, VRC inhibited cell growth by 38% and 75% at concentrations of 0.25 and 2 µg·mL^−1^, respectively (Figure 5A).

In contrast, 24 h biofilms formed in RPMI-1640 appeared more resistant to VRC treatment, exhibiting inhibition rates of 31% and 36% at concentrations of 0.25 and 2 µg·mL^−1^, respectively. VRC also inhibited cell growth under these conditions, although to a lesser extent than that observed in SDB. As observed for biofilm formation (Figure 1), glucose supplementation resulted in an intermediate susceptibility profile compared with biofilms formed in SDB and RPMI-1640. At 2 µg·mL^−1^ VRC, inhibition of metabolic activity reached 90%, 38%, and 48% in 24 h biofilms formed in SDB, RPMI-1640, and RPMI-1640 supplemented with glucose, respectively. Under the same conditions, planktonic cell growth was inhibited by 75%, 39%, and 56%, respectively.

A strong positive correlation was observed between the metabolic activity of 24 h biofilms and cell growth, with correlation coefficients (r) of 0.89, 0.78, and 0.92 for cultures grown in SDB, RPMI-1640, and RPMI-1640 supplemented with glucose, respectively.

**Figure 6:**
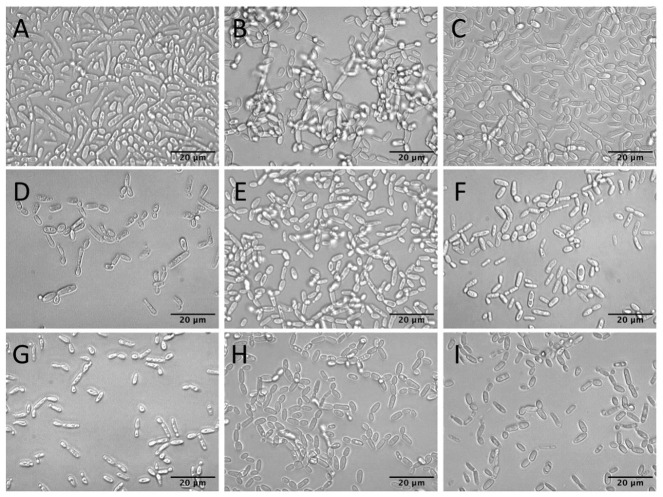
**Morphological characterization of 24 h *P. kudriavzevii* biofilms after exposure to VRC under different culture conditions.** Biofilms were initially formed for 24 h in SDB (**A**,**D**,**G**), RPMI-1640 medium (**B**,**E**,**H**) or RPMI-1640 supplemented with glucose (**C**,**F**,**I**), followed by an additional 24 h incubation in the absence (**A**–**C**) or presence of 1.0 µg·mL^−1^ VRC (**D**–**F**) or 2.0 µg·mL^−1^ VRC, as described in Section 2. Representative images were obtained by inverted light microscopy at 40× magnification. Scale bar = 20 µm.

Figure 6 shows the effect of VRC in reducing the number of cells within the structure of 24 h biofilms. A marked decrease in cell number was observed in 24 h biofilms developed in SDB in the presence of 1 and 2 µg·mL^−1^ VRC (Figure 6A,D,G). In contrast, 24 h biofilms formed in RPMI-1640 were also inhibited by VRC, although to a lesser extent. Consistent with the results observed during biofilm formation (Figure 1 and Figure 4), 24 h biofilms produced in RPMI-1640 supplemented with glucose exhibited an intermediate inhibition profile in response to VRC.

### 3.5. Growth Kinetics and Glucose Consumption Under VRC

**Figure 7:**
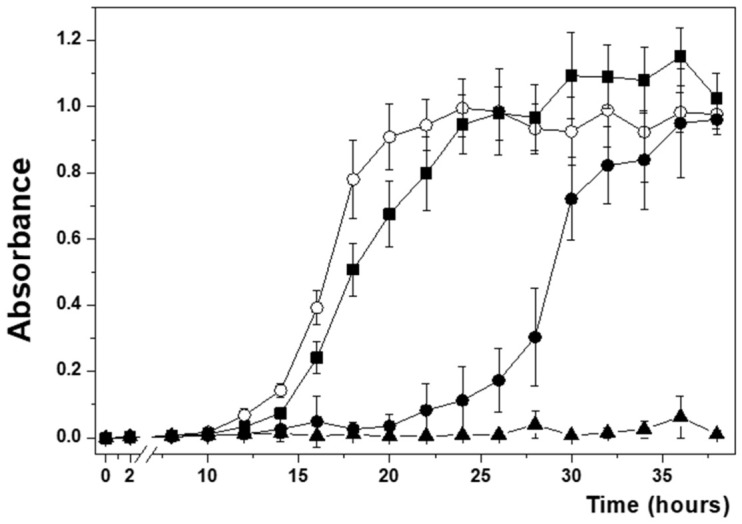
Time-course growth assay of *P. kudriavzevii* in the presence of VRC. *P. kudriavzevii* cells were cultured in SDB in the absence (○) or presence of 0.05 µg·mL^−1^ (■), 0.1 µg·mL^−1^ (•) or 0.5 µg·mL^−1^ (▲) VRC, as described in Section 2. Cell growth was monitored over 36 h by measuring optical density at 570 nm (OD570). Results are presented as mean ± standard deviation (SD).

After observing that the growth of *P. kudriavzevii* was more consistent and predictable in SDB medium and considering that VRC exhibited greater inhibitory activity under this condition, a growth time-course analysis in SDB was performed (Figure 7). Control cells exhibited a lag phase of approximately 10 h, whereas cells exposed to 0.05 and 0.1 µg·mL^−1^ VRC showed extended lag phases of 14 and 20 h, respectively, indicating that VRC delayed *P. kudriavzevii* growth. Complete growth inhibition was observed in the presence of 0.5 µg·mL^−1^ VRC. The profile of the exponential phase was also modified by VRC. Control cells exhibited a typical exponential growth profile between 12 and 20 h, with a growth coefficient of 0.0577 ± 0.0067. In contrast, cells growing in the presence of VRC showed significantly reduced growth coefficients. Cultures exposed to 0.05 and 0.1 µg·mL^−1^ VRC presented growth coefficients of 0.0366 ± 0.0067 and 0.00330 ± 0.00304, respectively. Furthermore, inhibition rates of 35.9 ± 14.1%, 94.1 ± 5.4%, and 99.0 ± 1.1% were determined for 0.05, 0.1, and 0.5 µg·mL^−1^ VRC, respectively.

The stationary phase began at 26 h in both the control group and cells treated with 0.05 µg·mL^−1^ VRC, whereas cells exposed to 0.1 µg·mL^−1^ VRC showed a delayed entry into the exponential phase, starting at 36 h.

The glucose content in SDB medium was also determined at 12, 20, and 28 h of cell growth (Table 1). Glucose consumption was observed during growth; however, at 12 h, no significant change in medium glucose content was detected. In the control group, a reduction of 95.5% and complete depletion of glucose were observed at 20 and 28 h, respectively. Reduced glucose consumption was observed in cells growing in the presence of 0.05 µg·mL^−1^ VRC, and virtually no glucose consumption was observed at 0.1 µg·mL^−1^ VRC. At 28 h, no detectable glucose remained in both the control group and in cultures treated with 0.05 µg·mL^−1^ VRC, according to the method used in this study.

Table 1 shows a clear correlation between glucose consumption and *P. kudriavzevii* growth in the presence of VRC. In the control group, glucose concentration decreased rapidly over time, reaching near-complete depletion at 20 h (0.91 g·L^−1^) and full depletion at 28 h. This profile was associated with intense yeast growth, reaching 92.7% at 20 h and 95.2% at 28 h, indicating active metabolism and efficient glucose utilization during the exponential phase.

Cells treated with 0.05 µg·mL^−1^ VRC showed delayed glucose consumption compared with the control group. At 20 h, residual glucose levels remained higher (4.97 g·L^−1^), while *P. kudriavzevii* growth was reduced to 69.0%. However, glucose was completely depleted and growth reached 98.7% at 28 h.

In contrast, cells exposed to 0.1 µg·mL^−1^ VRC exhibited minimal glucose consumption throughout the experiment. At 20 and 28 h, glucose concentrations remained high (17.61 and 16.78 g·L^−1^, respectively), corresponding to markedly reduced growth rates of 3.5% and 30.9%. These results indicate that this VRC concentration strongly reduced cellular metabolism, impairing glucose utilization and consequently inhibiting cell proliferation.

## 4. Discussion

The present study demonstrated that the antifungal activity of VRC against *P. kudriavzevii* is strongly influenced by the metabolic environment, particularly glucose availability. Our results indicate that fungal growth, biofilm formation, metabolic activity, and antifungal susceptibility are tightly interconnected and collectively affected by culture medium composition. The relationship between reduced glucose consumption and decreased cell proliferation suggests that inhibition of ergosterol biosynthesis by VRC may be associated with metabolic changes related to biofilm maintenance.

This interpretation is consistent with previous studies demonstrating that azole susceptibility is highly dependent on fungal metabolic activity and the energetic state of the cells [15,22,35]. A clear effect of culture medium composition was observed through the experiments, with results correlating with the high glucose concentration and nutrient-rich composition of SDB, which promote elevated metabolic flux, rapid proliferation, and increased extracellular matrix production [36,37,38]. Indeed, biofilms formed in SDB exhibited significantly greater biomass accumulation, metabolic activity, and cell density than those produced in RPMI-1640. In contrast, RPMI-1640, a chemically defined medium with physiological buffering capacity, supported reduced fungal proliferation and lower biofilm development. RPMI-1640 is currently recommended by CLSI and EUCAST for antifungal susceptibility testing because its standardized composition provides improved reproducibility and clinically relevant MIC interpretation [26,27,39].

The differences in VRC susceptibility observed among media support the idea that environmental conditions can affect azole pharmacodynamics and contribute to inter-study variability when non-standardized culture conditions are used [28,40,41]. Interestingly, although biofilms formed in SDB exhibited greater biomass accumulation and denser structural organization, VRC activity was markedly enhanced under these conditions, suggesting that biofilm biomass alone may not directly predict azole susceptibility under the conditions tested. This reinforces the concept that the physiological and metabolic state of biofilm cells plays a central role in determining antifungal response [37,42,43,44].

VRC inhibits lanosterol 14α-demethylase (Erg11), a key enzyme in the ergosterol biosynthesis pathway, thereby disrupting membrane synthesis, membrane integrity, and cellular homeostasis [15,45]. Since ergosterol production is associated with active cell growth, rapidly proliferating cells may require increased sterol synthesis, which could influence susceptibility to azoles [35,37]. Therefore, cells under glucose-rich conditions may exhibit increased demand for ergosterol biosynthesis, which could contribute to higher susceptibility to azole-induced stress. In contrast, nutrient-limited conditions can reduce metabolic demand and proliferation rates, supporting adaptive physiological states associated with increased antifungal tolerance [42,46]. In addition, nutrient-restricted conditions such as RPMI-1640 could induce adaptive responses associated with increased tolerance, including metabolic reprogramming, reduced proliferation, membrane remodelling, and activation of stress-response pathways [15,42].

The strong positive correlations observed between biofilm metabolic activity and fungal growth further support the existence of a close relationship between carbon metabolism and biofilm physiology. The highest correlation coefficients were detected in SDB and glucose-supplemented RPMI-1640, indicating that glucose availability is associated with a stronger correlation between cellular proliferation and metabolic activity. In contrast, the weaker correlation observed in RPMI-1640 suggests the presence of potentially heterogeneous physiological states, likely associated with nutrient limitation and physiological adaptation. These observations reinforce the concept that fungal biofilms are metabolically dynamic and physiologically heterogeneous structures rather than static biomass aggregates [44]. Biofilm-associated antifungal tolerance has usually been related to metabolic plasticity, stress adaptation, and the coexistence of subpopulations with distinct physiological states [37,42].

Microscopic analyses further demonstrated that environmental conditions strongly influenced biofilm architecture and stability. SDB promoted the formation of dense and highly adherent biofilms, whereas RPMI-1640 produced thinner and less structured biofilms. In addition, filamentous forms were observed in biofilms grown in RPMI-1640, suggesting that nutrient-restricted conditions may induce morphological and physiological adaptations associated with stress tolerance and altered antifungal susceptibility. Glucose supplementation partially restored biomass accumulation and structural organization in RPMI-1640, highlighting the central role of carbon availability in regulating adhesion, extracellular matrix production, and biofilm maturation [47,48,49]. However, increased biomass did not necessarily correlate with reduced VRC activity, indicating that susceptibility is more closely associated with metabolic state than with structural complexity alone.

Time-course experiments provided additional mechanistic evidence linking glucose metabolism to VRC effects. VRC prolonged the lag phase, reduced exponential growth rates, and inhibited fungal proliferation in a concentration-dependent manner. Importantly, increasing VRC concentrations progressively suppressed glucose consumption, directly correlating with delayed growth kinetics and reduced biomass accumulation. Cultures exposed to inhibitory VRC concentrations maintained elevated residual glucose levels throughout the experiment, indicating severe impairment of metabolic activity and carbon utilization. In contrast, cells exposed to subinhibitory concentrations partially recovered glucose consumption and resumed growth over time, suggesting possible adaptive responses to antifungal stress. These findings suggest that azole-induced membrane alterations may indirectly affect cellular nutrient uptake and metabolic processes, as previously described in the literature [46,50,51].

From a methodological perspective, our findings highlight the critical importance of standardized conditions for antifungal susceptibility testing (AFST). Variations in nutrient composition, glucose availability, nitrogen sources, and pH directly influence fungal physiology, biofilm formation, and azole activity, thereby affecting MIC determination [22,52]. Therefore, RPMI-1640 remains the reference medium recommended by CLSI and EUCAST because of its reproducibility and clinical applicability [26,27,28]. A limitation of this study is the use of a single reference strain (ATCC 6258), which may not fully represent the variability observed among clinical isolates. Antifungal susceptibility and biofilm-related responses can differ between strains; therefore, the findings should be interpreted within this context.

Collectively, our results support a model in which VRC activity against *P. kudriavzevii* is determined not only by its direct molecular target but also by the metabolic state of fungal cells and the nutritional environment in which biofilms develop. Glucose availability emerged as a central regulator of fungal growth, biofilm architecture, metabolic activity, and antifungal susceptibility. These findings support the concept that antifungal efficacy is context-dependent and highlight cellular metabolism as a key determinant of azole response in fungal biofilms [15,37]. Moreover, the results presented in this work suggest that metabolic pathways may represent potential contributors to antifungal response and warrant further investigation as potential therapeutic targets.

## 5. Conclusions

VRC activity against *P. kudriavzevii* was strongly influenced by glucose availability and culture conditions. SDB promoted higher biofilm development and metabolic activity, whereas RPMI-1640 resulted in reduced growth and biofilm formation. Although biomass was greater under glucose-rich conditions, antifungal susceptibility was more closely associated with metabolic activity and glucose utilization than with structural complexity. These results demonstrate that fungal metabolism is a key determinant of the VRC response in biofilms.

## Figures and Tables

**Table 1:** Effect of VRC on glucose consumption and growth of P. kudriavzevii in Time-course growth assay in SDB. Residual glucose concentrations (g·L^−1^) and growth percentages of P. kudriavzevii were determined during cultivation in SDB in the absence (Control) or presence of VRC (0.05 and 0.10 μg·mL^−1^). The initial glucose concentration at 0 h was 20 g·L^−1^. Glucose levels were quantified by HPLC after 12, 20 and 28 h of incubation. Data are presented as mean ± standard deviation (SD). * *p* < 0.05 compared with the control group.

	Control		VRC 0.05 µg·mL^−1^		VRC 0.10 µg·mL^−1^	
Time	Glucose	Growth	Glucose	Growth	Glucose	Growth
12 h	18.21 ± 2.3	6.9%	17.97 ± 2.1	3.3%	18.0 ± 2.1	1.9%
20 h	0.91 ± 0.1 *	92.7%	4.97 ± 0.6 *	69.0%	17.61 ± 2.3	3.5%
28 h	0.0	95.2%	0.0	98.7%	16.78 ± 2.3	30.9%

## Data Availability

The original contributions presented in this study are included in the article. Further inquiries can be directed to the corresponding author.
